# The Synergistic Effects of a Complementary Physiotherapeutic Scheme in the Psychological and Nutritional Treatment in a Teenage Girl with Type 1 Diabetes Mellitus, Anxiety Disorder and Anorexia Nervosa

**DOI:** 10.3390/children8060443

**Published:** 2021-05-25

**Authors:** Pelagia Tsakona, Vaios Dafoulis, Anastasios Vamvakis, Konstantina Kosta, Styliani Mina, Ioannis Kitsatis, Alexandra Hristara-Papadopoulou, Emmanuel Roilides, Kyriaki Tsiroukidou

**Affiliations:** 1 Department of Physiotherapy, Faculty of Health Sciences, International Hellenic University, Alexander Campus, 570 01 Thessaloniki, Greece; ptsakona@yahoo.gr (P.T.); alekpap@phys.teithe.gr (A.H.-P.); 2 Child-Adolescents’ Psychiatric Clinic, Hippokration General Hospital of Thessaloniki, 546 42 Thessaloniki, Greece; vadafoulis@yahoo.gr (V.D.); styliani.mina@gmail.com (S.M.); 3 Pediatric Endocrine Unit, 3rd Department of Pediatrics, Hippokration General Hospital of Thessaloniki, Aristotle University of Thessaloniki, 541 24 Thessaloniki, Greece; tvamvakis@yahoo.gr (A.V.); kkosta76@yahoo.gr (K.K.); ikitsatis@auth.gr (I.K.); roilides@auth.gr (E.R.)

**Keywords:** anorexia nervosa, anxiety disorder, physiotherapy, relaxation techniques, breathing exercise, eating disorder, psychiatric disorder, diet

## Abstract

Type 1 diabetes mellitus (T1DM) is a chronic disease that can affect the physical and mental health of children and adolescents, often leading to anxiety disorders with chronic activation of the hypothalamic axis (HPA). Moreover, a great proportion of adolescents with T1DM also demonstrate anorexia nervosa (AN), due to the increased preoccupation with food and the need to have an acceptable body image. Herein is described the first case study of an adolescent patient diagnosed with T1DM, anxiety disorder (AD), and AN. A 14-year-old girl with T1DM since the age of 12 years presented weight loss at age 13 years and 3 months and low body mass index (BMI), which did not improve despite dietary recommendations and adequate disease control. Additionally, she presented menstrual disorders at the age of 12 years and 11 months (menstrual age 12 years and 1 month). A psychological evaluation of the teenager was conducted using a semi-structured interview that assessed perceived stress, health status, quality of life, and depression. AD and AN were diagnosed and the patient initiated an intervention focusing on psychological health and nutrition and which incorporated physiotherapeutic relaxation sessions and breathing exercises. After 3 months of treatment, the patient’s BMI was increased, and a normal menstrual cycle was apparent. These results have since remained consistent. Stress leads to the appearance of AN and menstrual disorders. Therefore, physiotherapeutic programs could reduce stress and effectively ameliorate AN and AD.

## 1. Introduction

Type 1 diabetes mellitus (T1DM) is a chronic disease requiring adaptions to the daily routine of a child and their family, with an increased likelihood of disrupting family functioning [1,2,3], affecting their physical and psychological health, often leading to stress and depression [4,5]. Early exposure of children and adolescents to chronic stress augments the risk for anxiety disorders (ADs), altering hormonal secretion in the hypothalamic axis (HPA), with HPA-overactivity often resulting in disordered eating [6]. Although these endocrine imbalances are mostly a physiological adaptation to the low energy availability, they can also affect skeletal health and the psychosomatic status of the individual [7].

As a result, research suggests that quite often T1DM and anorexia nervosa (AN) coexist [8], complicating the need for integrated care, especially among adolescents. Most patients with AN and T1DM exhibit a low body mass index (BMI) and several micronutrient deficiencies, with zinc and vitamin D being the most frequent, followed by copper, selenium, and vitamin B1 [9,10]. AN is a complex eating disorder and one of the most life-threatening situations in an adolescent’s life [11,12]. Since the advent of the *Diagnostic and Statistical Manual of Mental Disorders, 5th Edition* (DSM-5) [13], and the broadening of the AN diagnostic criteria, a significant increase in the prevalence of AN has been observed [14,15]. Many different psychological, biological, environmental, familial, and socio-cultural factors have been implicated in the manifestation and course of AN [16,17,18].

The diagnosis and treatment of AN constitute a clinical challenge, requiring a comprehensive therapeutic team. To date, treatment methods applied in adolescent and adult patients with AN include drug and psychiatric treatment, medical nutrition therapy (MNT), inpatient care, and a variety of other “alternative” therapies including adjunctive yoga, family therapy, cognitive behavioral therapy, etc. [19,20].

According to Kolnes [21], body dissatisfaction and disturbances in bodily sensations are important components of the pathophysiology in patients with AN, and the application of physiotherapeutic approaches have been suggested [22] but rarely tested in the literature. Herein we report the case study of a 14-year-old adolescent girl with T1DM, AN, and AD diagnoses, in which a complementary physiotherapeutic intervention was applied.

## 2. Case-Study Report

Upon admission to the Pediatric Endocrine Unit situated at the 3rd Department of Pediatrics, Hippokration General Hospital of Thessaloniki, on 1 September 2016, a girl at the age of 11 years and 11 months was diagnosed for the first time with T1DM and an intercurrent symptom of low BMI. At the onset of Diabetes Mellitus (DM), her BMI ranged between the 3rd and 10th percentile.

The patient was treated by the DM expert team, which also involved three nutritionists and one pediatric psychologist, and was supported by the Child-Adolescent Psychiatric Clinic when necessary. At first, the DM management involved recurrent daily sessions for one week. Afterwards, the patient participated in sessions with a pediatric psychologist twice a month for the following six months, with each session lasting for forty-five minutes. Additionally, she participated in meetings with a dietitian twice a month, lasting for thirty minutes each. Thereafter, sessions were held once every two months, as the girl presented gradual improvement and her glycated haemoglobin (HbA1c) and BMI were stabilized.

The pediatric endocrinologist supervised the girl after her T1DM diagnosis at least once every three months on a programmed reassessment lasting for sixty minutes. One year later, at the age 12 years and 11 months the patient presented menstrual disorders (at the age of 12 years and 1 month) and increased stress. Then, the DM expert team rescheduled the sessions with the pediatric psychologist to twice a month again, and those with the nutritionist were held once/monthly. At the same time, the girl was evaluated by the pediatric psychiatrist and was diagnosed with AN.

Three months later, although the girl received nutritional and psychological intervention and had good glycemic control (low HbA1c levels without severe hypoglycemic episodes) during this period, her weight was significantly reduced (age of 13 years and 3 months). The pediatric endocrinologist suggested the initiation of physiotherapeutic exercises. The girl agreed, and an informed consent document was signed by her mother.

The physiotherapeutic sessions and the medical supervision were conducted at the Pediatric Endocrine Unit of General Hospital of Thessaloniki and did not involve any private medical expenses.

### 2.1. Nutritional Assessment

A detailed dietary assessment was performed using food-frequency questionnaires, previous day 24 h recalls, and a diet history. The assessment revealed, after the clinical examination, energy restriction leading to significantly low body weight (BW) (Table 1) according to her age, developmental trajectory, physical health, and sex. Moreover, the girl demonstrated a disturbed self-perceived body image with regard to her weight and shape. The patient exhibited a low BMI, low BW, and menstrual disorders, which quickly evolved to secondary amenorrhea. Furthermore, an intense fear of excess BW and fatness was apparent from the interviews, although she was in fact underweight.

### 2.2. Psychiatric Assessment

The girl and her family were psychologically evaluated by a pediatric psychiatrist, using a diagnostic semi-structured interview based on the Kiddie Schedule for Affective Disorders and Schizophrenia (K-SADS) questionnaire for school-age children aged 6–18 years, validated for the Greek population [23].

The K-SADS questionnaire [23] is a semi-structured interview with useful questions adapted to the language of the parent and child. The goal of the K-SADS questionnaire [23] is to collect information from both the parents and the child, referring to the current diagnosis, the disorders for which medications might have been provided, the history of the illness and diagnoses, and the time period of the illness. The child’s reporting accuracy along with the parental ignorance about the symptoms was evaluated by the clinical judgment of the pediatric psychiatrist. For increased reliability, the administration of the K-SADS questionnaire [23] was performed by two therapists (the pediatric psychiatrist and the psychologist) at the same time, in a first interview when the girl was age 13 years and 3 months.

In this interview, a unanimous diagnosis of generalized anxiety disorder (AD) (F40 v ICD-10) was made. The dominant symptoms were excessive stress for a prolonged period of time, even for petty things, and worrying about her illness; this stress was accompanied by tension and an inability to relax. The girl also exhibited psychosomatic symptoms, including frequent headaches.

During this first interview, the adolescent completed the State-Trait Anxiety Inventory Questionnaire for children, which has been validated for the Greek population, consisting of two scales: The A-State Anxiety Scale and the A-Trait Anxiety Scale [24]. The A-State Anxiety Scale consists of 20 questions and is designed to measure the subjective feelings of nervousness, fear, and anxiety, with variable intensity, fluctuating depending on the situation [24]. The A-Trait Scale [24] consists of 20 questions and measures individual differences in the way children experience stress in their daily lives. The State-Trait Anxiety Inventory Questionnaire confirmed again the diagnosis of generalized AD.

### 2.3. Physiotherapeutic Assessment

The patient and her mother were interviewed by a pediatric physiotherapist specialized in children and adolescents (P.T.). Information on participation in previously implemented relaxation techniques, diaphragmatic breathing exercises, or in any sports was based on self-reports during a face-to-face first interview that lasted for one hour. Before the intervention, the patient did not exercise or participate in any sports.

The purpose of the intervention in the present case-study was to evaluate the effectiveness of a stress management program, including morning aerobic therapeutic exercises (activating and strengthening muscles; increasing blood flow throughout the body; improving body flexibility, balance, and general lung function; and preventing musculoskeletal problems). In addition, the program included evening relaxation techniques (progressive muscle relaxation; pressure and self-massage on her palms, soles, and on her body, along the path of meridians at specific acupuncture points; and diaphragmatic breathing).

## 3. Therapy

### 3.1. Psychological Treatment

The National Institute for Health and Care Excellence (NICE) guidelines were used for the development of an age-appropriate therapeutic scheme [25], using AN-focused family therapy (FT-AN) in weekly sessions. The FT-AN, which included psychoeducation concerning matters related to nutrition and malnutrition, was aimed at building independence, enhancing self-efficacy, explaining the risks of malnutrition and being underweight, and encouraging reaching a healthy BW and healthy eating, [25]. The patient was asked to apply positive health behaviors, and she was supported for the next three months with educational material consisting of written instructions on healthy lifestyle practices.

### 3.2. Medical Nutrition Therapy

Medical nutrition therapy (MNT) was based on the Junior MARSIPAN (Management of Really Sick Patients under 18 with Anorexia Nervosa) [26] and the Academy of Nutrition and Dietetics [27] guidelines. The MNT focused on avoiding the refeeding syndrome, improving weight gain, and promoting healthy eating, using the Recovery from Eating Disorders for Life (REAL) Food Guide [28]. The use of a nasogastric tube was avoided.

### 3.3. Physiotherapy Intervention

The girl was trained according to a physiotherapeutic exercise protocol with the additional help of an educational DVD (digital video disc). The DVD and therapeutic protocol were designed (P.T. and A.H.P.) at the Department of Physiotherapy of International Hellenic University in Thessaloniki. The physiotherapeutic exercise protocol followed a script prepared by the pediatric physiotherapist.

Training took place in a dedicated and quiet facility in the Pediatric Endocrine Unit and was performed in two sessions in the first week of the intervention. The first session included a morning moderate-intensity therapeutic exercise program in five basic positions (supine, sideways, prone, four-legged, and sitting), always combined with diaphragmatic breathing techniques and lasting twenty minutes in total [22]. The second session included an evening physiotherapeutic relaxation program of diaphragmatic breathing, progressive muscle relaxation, stretching exercises for the main muscles of the body, and pressure on the palm, soles, and specific acupuncture points in a similar twenty minutes’ duration as the first session.

In addition, a DVD was provided to the girl with three videos describing the morning and evening exercise schedule, a brochure containing the exact description of the physiotherapeutic exercise protocol, and the breathing device (a TriFlo) as a gift with which to practice daily diaphragmatic breathing in a sitting position. She was asked to perform these techniques every day—morning and evening—for twelve weeks. The girl responded positively to the physiotherapeutic program. She was very cooperative, happy, and excited.

Her compliance was evaluated once a week with a telephone call and by two personal interviews on monthly follow-ups at the hospital. During the telephone calls, the girl was encouraged to continue the program. Two weeks after the beginning of the intervention and in all subsequent sessions until the end of three-month period, the girl declared that she performed the therapeutic program daily according to the given instructions. Additionally, she complied with the respiratory exercises for diaphragmatic breathing and with the self-massage techniques on the acupuncture points on most days during all of this period. The girl asserted that this intervention was helping her to relax from stress, to lengthen the muscles and joints from the daily strain, and to maintain herself in good physical fitness. She was feeling strong, healthy, calm, and full of energy. The girl was informed that all measurements (completions of psychometric tools measuring stress and lifestyle, BMI, BW) would be repeated after a three-month period.

## 4. Results

Three months after the initiation of the therapeutic scheme, on 28 March 2018, the girl was re-evaluated with the use of the K-SADS-P questionnaire, at the age of 13 years and 6 months. At that time, she was no longer suffering from generalized anxiety disorder. The post-intervention State-Trait Anxiety Inventory questionnaire responses showed an increase in positive emotions, no negative feelings or reporting of low self-esteem, and improvement in anxiety disorder with much less tension or inability to relax, confirming the result of the K-SADS-P diagnostic interview.

The BMI of the girl reached “normal” values, her menstrual cycle was restored, and her HbA1c was stabilized. Moreover, she gained 3.1 kg of body weight during the first 3 months after the initiation of the multidisciplinary intervention (on 11 January 2018, the girl weighed 47.6 kg, and after 3 months, on 28 March 2018, she weighed 50.7 kg).

Changes in stature, body weight, BMI, and HbA1c before and after the initiation of the multidisciplinary intervention are as presented in Table 1.

**Figure 1:**
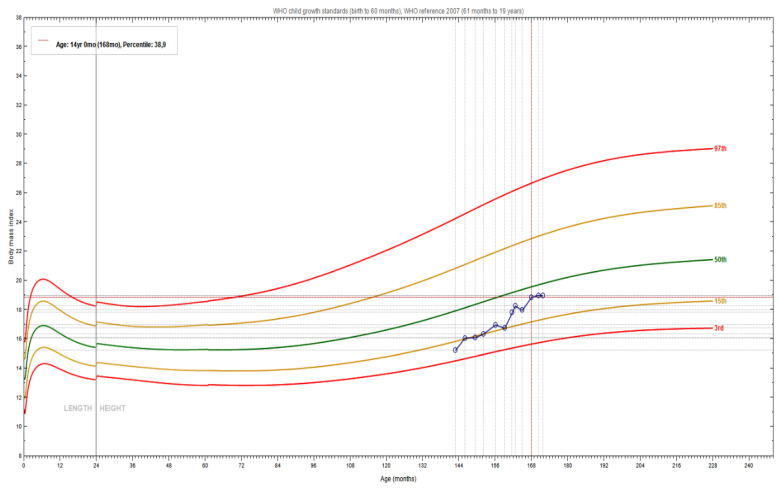
Improvement in the body mass index of the patient during the intervention.

Figure 1 details BMI improvements during the intervention period. With regard to the psychological outcomes, after the intervention the girl also appeared less fearful of excessive body weight and fatness. Today, she continues to follow the intervention program.

**Figure 2:**
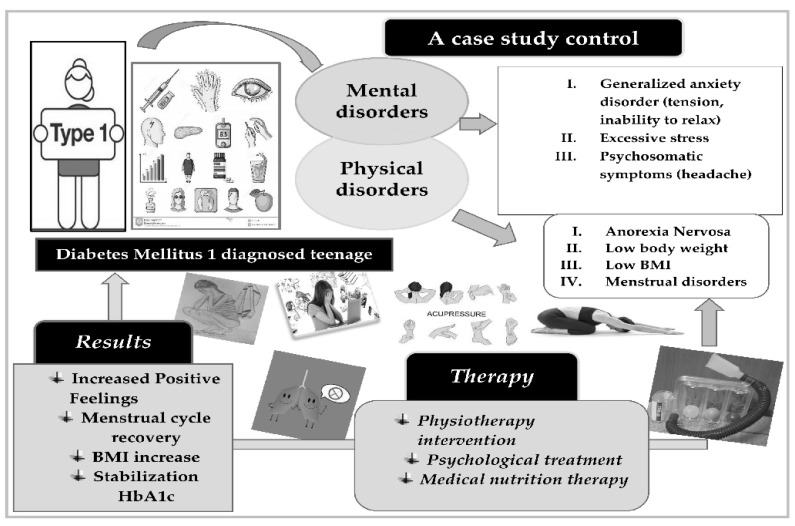
A description and the results of a combined therapeutic intervention in the mental and physical health of a teenage girl diagnosed with T1DM, AN, AD, and menstrual disorders.

The girl and her family considered the physiotherapeutic program as very interesting, useful, and pleasant. Three months after the intervention, she declared improved well-being and better family relationships as well as improved mental and physical health. She stated that the combined therapeutic intervention (psychological, nutritional, and physiotherapeutic) helped her to see her life positively and with increased self-confidence in managing diabetes and every other difficulty in her life (Figure 2).

## 5. Discussion

The present case study verified the frequent co-occurrence of eating disorders with T1DM and showed how incorporating daily physiotherapeutic sessions as part of usual care can improve health outcomes and growth among adolescents with concurrent AN and T1DM diagnoses.

The daily routine of constant food monitoring and of calculating carbohydrate intake and insulin doses imposes a new reality in the life of an individual with T1DM, making them more prone to AN [29]. This new situation is additionally burdened with a regular reassessment of insulin needs, particularly when factors that influence it are involved, such as illness, exercise, stress, etc. Adolescents with T1DM often demonstrate elevated body fat levels despite the adoption of a healthy diet [30], which in turn might propel excessive stress and body image disorders. Significant fluctuations in glucose levels beyond the risk of immediate complications cause psychological stress, constituting an additional metabolic stressor. Moreover, numerous studies have shown that parental discomfort leads to more negative and fewer positive interactions with adolescents [31,32], revealing that family functioning also plays a key role in the establishment and development of AN [33,34].

AN is the most studied and well-known eating disorder (ED) that usually develops during adolescence. AN is a psychiatric disorder characterized by deteriorated body image, persistent eating restriction and low body weight as well as the structural and functional brain alterations that are associated with this psychopathology [13,35]. In 2013, the American Psychiatric Association revised the diagnostic criteria for AN, making the criteria about the body weight less restrictive, while removing the criterion for amenorrhea in order to make the criteria gender-neutral [13,36]. The hypothalamic–pituitary–adrenal (HPA) axis is in a state of chronic stimulation in at least one-third of women with AN [36]. As a result, AN is associated with endocrine dysfunction, including HPA dysfunction and changes in adiponectin and appetite-regulating hormone levels [37].

On the other hand, in AN, stress and psychological disorders can affect the HPA and affect hormonal regulation (increase in cortisol levels, stress hormone). Dysregulation of the HPA might persist in women with AN even after the weight gain, which suggests that recovery from AN is not complete despite weight gain, or that the HPA might be involved in disease pathogenesis [38,39].

Since AN is often characterized by excessive exercise engagement, any form of exercise is excluded from the therapeutic models proposed for treating patients with AN, in fear of aggravating the symptoms [13,40]. In a systematic review [40], it was shown that physiotherapy interventions have a large effect on ED outcomes as compared with the usual care or waitlist for treatment. This effect was also extended to the quality of life and disordered eating of participants. Therefore, it appears that physiotherapy has a clinical significance in the management of EDs and can be used to improve body image [41]. The exact mechanism of physiotherapy in improving ED outcomes is not clear; however, it has been reported that a great proportion of patients with EDs exhibit low self-esteem and problems with relaxation [41,42]. Thus, it is possible that physiotherapy acts directly on these problems by relieving stress and improving self-esteem. Moreover, physiotherapy acts on other outcomes, including functional recovery [43] and improved breathing [22]. The latter particularly has been associated with “heavy” and difficult thoughts and suppressed feelings. According to Probst [44], physiotherapists are equipped with the necessary skills to address these issues among patients with AN, improving the distorted body image and stress typically observed in AN.

In addition, studies have shown that the application of simple daily exercises focusing on strength, balance, and flexibility, in combination with diaphragmatic breathing exercises, improves physical condition, health, concentration, and mental state [45,46,47]. Stress is reduced post-exercise through the release of β-endorphin and β-lipotrophin, substances that are natural painkillers and are normally produced in the brain and cause a sense of wellbeing while lowering cortisol levels (primary hormone of stress) [48]. The use of a respiratory device (such as TriFlo, flutter, spirometer, as well as various musical instruments, such as flute, harmonica) increases the effectiveness of therapeutic exercise and pulmonary function [49].

The number of studies investigating the effects of physiotherapeutic exercise on adolescents with T1DM, AD, and AN are limited. In the present case study, a 14-year-old girl with all these diagnoses initiated a therapeutic scheme focusing on psychological health and nutrition with incorporated physiotherapeutic sessions. After 12 months of intervention, improvement and stability in AD, AN, menstrual disorders, weight gain, and BMI were noted.

## 6. Conclusions

In summary we described the case study of a pediatric patient with T1DM, AD, and AN which successfully improved with physiotherapeutic techniques. The appearance of AN and menstrual disorders, especially in adolescents and children with chronic illnesses, is associated with stress. Hence, the key to resolving these difficult situations is to deal with the stress. Physiotherapeutic programs can help in this regard if they are sufficiently well designed, especially for children and adolescents.

## Figures and Tables

**Table 1:** The scores of BMI and HbA1c before and after intervention.

Date (dd/mm/y)	Height (cm)	Body Weight (kg)	Age (yrs, mos)	BMI (kg/m^2^)	HbA1c (%)
Prior to treatment					
01/09/2016	162.2	40.0	11 yrs, 11 mos	15.2	14.0
06/12/2016	165.0	43.7	12 yrs, 3 mos	16.1	5.8
21/03/2017	166.0	44.3	12 yrs, 5 mos	16.1	6.4
13/06/2017	166.0	45.0	12 yrs, 8 mos	16.3	6.9
10/10/2017	168.6	48.2	13 yrs, 0 mos	16.9	6.8
Start of treatment					
11/01/2018	168.7	47.6	13 yrs, 3 mos	16.7	6.8
After treatment					
28/03/2018	168.8	50.7	13 yrs, 6 mos	17.8	6.9
27/04/2018	168.8	52.0	13 yrs, 7 mos	19.3	6.5
06/07/2018	169.4	51.5	13 yrs, 8 mos	18.0	6.3
09/10/2018	169.6	54.2	14 yrs, 0 mos	18.9	6.7
18/12/2018	169.8	54.7	14 yrs, 2 mos	19.0	6.2
31/01/2019	170.4	55.0	14 yrs, 4 mos	18.9	6.3

BMI, body mass index; HbA1c, glycosylated hemoglobin; yrs, years; mos, months.

## Data Availability

All data regarding the study are presented in the manuscript text.
